# Fat Accumulation and Elevated Free Fatty Acid Are Associated With Age-Related Glucose Intolerance: Bunkyo Health Study

**DOI:** 10.1210/jendso/bvad164

**Published:** 2023-12-20

**Authors:** Hitoshi Naito, Hideyoshi Kaga, Yuki Someya, Hiroki Tabata, Saori Kakehi, Tsubasa Tajima, Naoaki Ito, Nozomu Yamasaki, Motonori Sato, Satoshi Kadowaki, Daisuke Sugimoto, Yuya Nishida, Ryuzo Kawamori, Hirotaka Watada, Yoshifumi Tamura

**Affiliations:** Department of Metabolism & Endocrinology, Juntendo University Graduate School of Medicine, Juntendo University, Tokyo 113-8421, Japan; Department of Metabolism & Endocrinology, Juntendo University Graduate School of Medicine, Juntendo University, Tokyo 113-8421, Japan; Sportology Center, Juntendo University Graduate School of Medicine, Juntendo University, Tokyo 113-8421, Japan; Sportology Center, Juntendo University Graduate School of Medicine, Juntendo University, Tokyo 113-8421, Japan; Sportology Center, Juntendo University Graduate School of Medicine, Juntendo University, Tokyo 113-8421, Japan; Department of Metabolism & Endocrinology, Juntendo University Graduate School of Medicine, Juntendo University, Tokyo 113-8421, Japan; Department of Metabolism & Endocrinology, Juntendo University Graduate School of Medicine, Juntendo University, Tokyo 113-8421, Japan; Department of Metabolism & Endocrinology, Juntendo University Graduate School of Medicine, Juntendo University, Tokyo 113-8421, Japan; Department of Metabolism & Endocrinology, Juntendo University Graduate School of Medicine, Juntendo University, Tokyo 113-8421, Japan; Department of Metabolism & Endocrinology, Juntendo University Graduate School of Medicine, Juntendo University, Tokyo 113-8421, Japan; Department of Metabolism & Endocrinology, Juntendo University Graduate School of Medicine, Juntendo University, Tokyo 113-8421, Japan; Department of Metabolism & Endocrinology, Juntendo University Graduate School of Medicine, Juntendo University, Tokyo 113-8421, Japan; Department of Metabolism & Endocrinology, Juntendo University Graduate School of Medicine, Juntendo University, Tokyo 113-8421, Japan; Sportology Center, Juntendo University Graduate School of Medicine, Juntendo University, Tokyo 113-8421, Japan; Department of Metabolism & Endocrinology, Juntendo University Graduate School of Medicine, Juntendo University, Tokyo 113-8421, Japan; Sportology Center, Juntendo University Graduate School of Medicine, Juntendo University, Tokyo 113-8421, Japan; Department of Metabolism & Endocrinology, Juntendo University Graduate School of Medicine, Juntendo University, Tokyo 113-8421, Japan; Sportology Center, Juntendo University Graduate School of Medicine, Juntendo University, Tokyo 113-8421, Japan

**Keywords:** insulin resistance, disposition index, β-cell function, older adults

## Abstract

**Context:**

Older adults have a high prevalence of new-onset diabetes, often attributed to age-related decreases in insulin sensitivity and secretion. It remains unclear whether both insulin sensitivity and secretion continue to deteriorate after age 65.

**Objective:**

To investigate the effects of aging on glucose metabolism after age 65 and to identify its determinants.

**Methods:**

This cross-sectional study involved 1438 Japanese older adults without diabetes. All participants underwent a 75-g oral glucose tolerance test (OGTT). Body composition and fat distribution were measured with dual-energy X-ray absorptiometry and magnetic resonance imaging. Participants were divided into 4 groups by age (65-69, 70-74, 75-79, and 80-84 years) to compare differences in metabolic parameters.

**Results:**

Mean age and body mass index were 73.0 ± 5.4 years and 22.7 ± 3.0 kg/m^2^. The prevalence of newly diagnosed diabetes increased with age. Fasting glucose, fasting insulin, the area under the curve (AUC)-insulin/AUC-glucose and insulinogenic index were comparable between groups. AUC-glucose and AUC-insulin during OGTT were significantly higher and Matsuda index and disposition index (Matsuda index · AUC-insulin/AUC-glucose) were significantly lower in the age 80-84 group than in the age 65-69 group. Age-related fat accumulation, particularly increased visceral fat area (VFA), and elevated free fatty acid (FFA) levels were observed. Multiple regression revealed strong correlations of both Matsuda index and disposition index with VFA and FFA.

**Conclusion:**

Glucose tolerance declined with age in Japanese older adults, possibly due to age-related insulin resistance and β-cell deterioration associated with fat accumulation and elevated FFA levels.

The high incidence of new-onset diabetes in older adults is a well-known and pressing issue, especially in countries like Japan, which has the highest population aging rate in the world [1]. Older adults have reduced β-cell function and increased insulin resistance, leading to a higher prevalence of diabetes [2-7]. For example, a large study of Chinese adults reported a significant reduction in early-phase insulin secretion, as measured with the insulinogenic index, in older individuals (>60 years) compared with younger (20-39 years) and middle-aged (40-59 years) counterparts [4]. Furthermore, insulin sensitivity assessed through the meal and intravenous glucose tolerance test was lower in older adults than in young adults [6]. Despite these findings, it remains uncertain whether these factors deteriorate further after age 65, potentially exacerbating glucose tolerance. If so, it also remains uncertain what causes the deterioration in insulin sensitivity and β-cell function. Reduced muscle mass and increased body adiposity associated with aging could contribute to the deterioration in insulin sensitivity [8, 9]. In addition, chronic exposure to high concentrations of free fatty acids (FFAs) could be toxic for β cells [10]. Unraveling this mechanism could contribute to establishing an effective strategy for the prevention of new-onset diabetes mellitus in older adults.

The purpose of this study was to elucidate the effects of aging on glucose metabolism in older adults after age 65 and to identify its determinants. In this study, we assessed glucose tolerance, insulin secretion, and insulin sensitivity in community-dwelling older adults without a history of diabetes using a 75-g oral glucose tolerance test (OGTT).

## Methods

### Study Design and Participants

This cross-sectional study used the baseline data from the Bunkyo Health Study [11]. Briefly, we recruited 1629 individuals aged 65 to 84 years living in Bunkyo-ku, an urban area in Tokyo, Japan from October 15, 2015, to October 1, 2018. Among the Bunkyo Health Study participants, we only included those who had not been diagnosed with diabetes and had available 75-g OGTT data. Among the study participants, 187 were diagnosed with diabetes and 75-g OGTT data were unavailable for 4 participants. The remaining 1438 participants were included in this study.

The study protocol was approved by the ethics committee of Juntendo University in November 2015 (Nos. 2015078, and M15-0057). Briefly, subjects were evaluated over 2 days. On the first day, we evaluated cognitive function, muscle strength, and physical performance. On the second day, after an overnight fast, we evaluated body weight and composition with dual-energy X-ray absorptiometry (DXA), abdominal fat distribution with magnetic resonance imaging (MRI), and glucose tolerance with a 75-g OGTT. This study was carried out in accordance with the principles outlined in the Declaration of Helsinki. All participants gave written informed consent and were informed that they had the right to withdraw from the study at any time.

### Procedure for a 75-g OGTT and Definition of Glucose Tolerance

All participants underwent a 75-g OGTT. In this study, a standard 75-g OGTT was carried out in the morning after an overnight fast. The participants are instructed to eat a well-balanced diet for 3 days prior to the test, to refrain from a low-carbohydrate diet. Blood samples were collected immediately before, as well as 30, 60, 90, and 120 minutes after ingestion of glucose. We measured hemoglobin A1c (HbA1c) on the same day. According to the diagnostic criteria for diabetes of the Japan Diabetes Society, diabetes mellitus was defined as fasting plasma glucose (FPG) ≥ 126 mg/dL and/or a 2-hour glucose level after the 75-g OGTT ≥ 200 mg/dL and/or HbA1c ≥ 6.5% [12]. Normal glucose tolerance (NGT) was defined as FPG < 110 mg/dL, a 2-hour glucose level after the 75-g OGTT < 140 mg/dL, and hemoglobin A1c < 6.5%. The remaining participants were defined as having prediabetes.

### Evaluation of β-Cell Function, Insulin Sensitivity, and Insulin Clearance

The homeostasis model assessment of insulin resistance index (HOMA-IR) was calculated as [fasting serum insulin (µU/mL) · FPG (mg/dL)/405]. The Matsuda index was calculated using the following equation: [10 000/square root of (FPG [mg/dL] · fasting insulin [µU/mL]) · (mean glucose [mg/dL] · mean insulin during OGTT [µU/mL])] [13]. The insulinogenic index, reflecting early-phase glucose-dependent insulin secretion, was calculated using the following equation: [change in insulin/change in glucose from 0 to 30 minutes] [14]. Insulin secretion in response to blood glucose levels was also evaluated based on the ratio of the area under the curve (AUC) for insulin to glucose during OGTT (AUC-insulin/AUC-glucose). β-cell function was evaluated based on the disposition index (Matsuda index · AUC-insulin/AUC-glucose) [15]. Adipose tissue insulin resistance index (Adipo-IR) was calculated as [fasting insulin (µU/mL) · fasting FFA (mEq/L)] [16]. Serum insulin levels were measured by chemiluminescent enzyme immunoassay (FUJIREBIO Inc., Tokyo, Japan, Cat# 291290, RRID: AB_3065260). FFA was measured by an enzymatic method (SEKISUI Medical Co., Ltd, Tokyo, Japan). Insulin clearance was calculated as [C-peptide (ng/mL)/insulin (μU/mL)].

### Measurement of Visceral Fat Area and Subcutaneous Fat Area

Intra-abdominal fat area and subcutaneous fat area were measured with a 0.3-T MRI scanner (AIRIS Vento, Hitachi, Japan) as described previously [11]. Briefly, T1-weighted transaxial scans were obtained. Intra-abdominal fat area and subcutaneous fat area at the fourth and fifth lumbar interspaces were measured as described previously using specialized image analysis software (AZE Virtual Place, Canon Medical Systems Corporation, Japan).

### Other Measurements

Appendicular skeletal muscle mass (ASM) was measured using DXA (Discovery DXA System, Hologic, Tokyo, Japan). Skeletal muscle mass index (SMI) was calculated by dividing ASM by height squared in meters (kg/m^2^). Handgrip strength was measured twice on each side using a hand grip dynamometer (T.K.K.5401, Takei Scientific Instruments Co., Ltd., Japan). We used the average of the maximum values on each side for handgrip strength. Physical activity was evaluated using the International Physical Activity Questionnaire, which assesses different types of physical activity, such as walking and both moderate- and high-intensity activities [17, 18]. Nutritional status was evaluated using a brief self-administered diet history questionnaire that contained 58 items about fixed portions and food types [19, 20]. Hypertension was defined as systolic blood pressure ≥ 140 mmHg, diastolic blood pressure ≥ 90 mmHg or current use of antihypertensive medications. Dyslipidemia was defined as low-density lipoprotein cholesterol ≥ 140 mg/dL, high-density lipoprotein cholesterol < 40 mg/dL, triglycerides ≥ 150 mg/dL, or current use of lipid-lowering agents. Cardiovascular disease was defined by the World Health Organization as ischemic heart disease, cerebrovascular disease, or peripheral arterial disease. Sarcopenia was defined as weak handgrip strength and low SMI based on the definition of the Asian Working Group for Sarcopenia (AWGS) 2019 [21].

### Statistical Analysis

We classified participants by age (65-69 years, 70-74 years, 75-79 years, and 80-84 years) and divided them into 3 groups (NGT, prediabetes, and diabetes). We used IBM SPSS Statistics for Windows, version 28.0. (IBM Corp., Armonk, NY, USA) for statistical analysis. Data are presented as means ± SD, means ± SE, numbers (%), and medians (interquartile range), as appropriate. To approximate the normal distribution, log-transformed values were used in the analysis, as appropriate. Differences in means and proportions were tested using one-way analysis of variance (ANOVA) and chi-square tests. The Jonckheere-Terpstra trend test was used to investigate trends with aging. Differences of AUC-insulin/AUC-glucose, insulinogenic index, Matsuda index and disposition index were tested using ANCOVA with adjustment for sex, hypertension, dyslipidemia, cardiovascular disease, and sarcopenia. The relationship between the Matsuda index, disposition index, and various metabolic parameters was assessed with Pearson or Spearman correlation coefficients, as appropriate. Multiple regression analysis was performed to determine the independent contribution of insulin resistance and β-cell function. In this study, 2 models were used in regression analyses. Model 1 adjusted for age, sex, and visceral fat area (VFA). Model 2 adjusted for variables in Model 1 plus subcutaneous fat area (SFA), ASM, handgrip strength, FFA, adiponectin, C-reactive protein (CRP), and physical activity. All statistical tests were two-sided with a 5% significance level.

## Results

The characteristics and metabolic parameters of the study participants are shown in Table 1. There were no differences in the proportion of men and women across groups. The proportion of participants with normal glucose tolerance was significantly lower in the older group, while the proportion of participants with diabetes was significantly higher in the older group. Although physical activity levels and sedentary time were comparable among the groups, energy intake was higher in the older group, with carbohydrate intake significantly higher in the group aged 80 to 84 years than in the 2 groups aged under 75 years, and protein intake was significantly higher in the 2 groups older than 75 years than in the 2 groups younger than 75 years. Body weight was lower, and height was relatively lower in the older group, resulting in higher body mass index (BMI) in the older group. In terms of body composition, percent body fat was higher in the 2 groups older than 75 years than in the age group 65 to 69 years. Although ASM was significantly lower in the 2 groups aged over 75 years than in the 2 groups aged under 75 years, SMI was comparable among the groups. Handgrip strength was significantly higher in the age group aged 65 to 69 years than in the 3 groups aged over 70 years and significantly higher in the age group 70 to 74 years than in the 2 groups aged over 75 years. VFA was significantly higher in the age 80 to 84 age group than in the 65 to 69 age group, while SFA was comparable among the groups. The prevalence of sarcopenia, hypertension, and cardiovascular disease increased with age. Trend analysis also showed similar age-related changes in these parameters except for physical activity level and alcohol intake.

**Table 1.** Clinical characteristics and metabolic parameters of the study participants

Age group (years)	65-69	70-74	75-79	80-84	*P* value	*P* for trend
Number	465	422	326	225	—	—
Age (years)	67.0 ± 1.4	71.9 ± 1.5	76.8 ± 1.5	81.7 ± 1.4	—	—
Female sex (%)	60.6	57.3	63.1	60.1	.436	—
Height (cm)	159.7 ± 8.1	158.6 ± 8.3	155.9 ± 8.6^*,^*^†^*	154.3 ± 9.1^*,^*^†^*	<.001	<.001
Body weight (kg)	57.6 ± 10.5	57.0 ± 10.2	55.6 ± 9.9*	55.3 ± 9.3*	.006	.002
Body mass index (kg/m^2^)	22.9 ± 3.0	23.0 ± 2.9	23.2 ± 3.3	23.6 ± 2.9*	.034	.007
Percent body fat (%)	22.3 ± 6.1	22.5 ± 6.0	23.5 ± 6.2*	24.2 ± 6.0^*,^*^†^*	<.001	<.001
Skeletal muscle mass index (kg/㎡)	7.1 ± 1.1	7.1 ± 1.1	7.0 ± 1.0	7.0 ± 0.9	.195	.249
Appendicular skeletal muscle mass (kg)	18.4 ± 4.3	18.1 ± 4.1	17.2 ± 3.8^*,^*^†^*	16.9 ± 3.4^*,^*^†^*	<.001	<.001
Handgrip strength (kg)	27.7 ± 7.4	26.4 ± 7.0*	24.1 ± 6.4^*,^*^†^*	22.9 ± 5.8^*,^*^†^*	<.001	<.001
Sarcopenia (n; %)	17 (3.7)	42 (10.0)	46 (14.1)	57 (25.3)	<.001	.042
Subcutaneous fat area (cm^2^)	147.6 ± 55.9	146.0 ± 57.5	152.9 ± 62.7	154.6 ± 58.1	.187	.161
Visceral fat area (cm^2^)	71.5 ± 39.4	76.3 ± 36.7	77.1 ± 38.1	83.8 ± 33.9*	.001	<.001
Normal glucose tolerance (n; %)	323 (69.5)	227 (53.8)	167 (51.2)	107 (47.6)	<.001	.042
Prediabetes (n; %)	107 (23.0)	148 (35.1)	120 (36.8)	76 (33.8)	<.001	.497
Diabetes (n; %)	35 (7.5)	47 (11.1)	39 (12.0)	42 (18.7)	<.001	.042
Hypertension (n; %)	247 (53.1)	280 (66.4)	230 (70.6)	171 (76.0)	<.001	.042
Dyslipidemia (n; %)	268 (57.6)	263 (62.3)	211 (64.7)	134 (59.6)	.202	.497
Cardiovascular diseases (n; %)	26 (5.6)	29 (6.9)	27 (8.3)	27 (12.0)	.024	.042
Physical activity level (MET·hour/week)	30.5 (17.9-51.1)	34.0 (19.3-59.1)	31.8 (16.5-57.8)	24.8 (13.2-43.8)	.073	.028
Sedentary time (hours)	6.0 ± 3.4	5.8 ± 3.4	6.0 ± 3.9	6.0 ± 3.8	.848	.321
Dietary intake (kcal/day)	1943.6 ± 576.4	1919.7 ± 609.9	2023.9 ± 625.2	2042.3 ± 597.9	.021	.019
Carbohydrate (g/day)	239.7 ± 82.4	237.1 ± 83.8	247.8 ± 85.8	258.9 ± 84.1^*,^*^†^*	.008	.006
Protein (g/day)	79.2 ± 26.4	80.6 ± 31.3	88.9 ± 33.3^*,^*^†^*	88.6 ± 31.7^*,^*^†^*	<.001	<.001
Fat (g/day)	61.1 ± 20.5	60.3 ± 22.1	64.5 ± 23.3	62.2 ± 21.6	.053	.140
Alcohol (g/day)	2.2 (0.0-21.0)	1.3 (0.0-18.2)	0.3 (0.0-10.6)	0.6 (0.0-14.3)	.192	.001
Fasting plasma glucose (mg/dL)	96.2 ± 11.6	97.7 ± 10.8	96.9 ± 11.3	97.0 ± 11.3	.240	.218
Fasting serum insulin (µU/mL)	4.55 ± 3.17	4.76 ± 2.93	4.93 ± 3.34	5.03 ± 2.99	.190	.005
Fasting C-peptide (ng/mL)	1.36 ± 0.60	1.41 ± 0.58	1.44 ± 0.58	1.52 ± 0.62*	.011	<.001
Fasting free fatty acids (mEq/L)	475.3 ± 189.3	509.4 ± 197.9*	548.8 ± 213.3^*,^*^†^*	577.5 ± 217.1^*,^*^†^*	<.001	<.001
HbA1c (%)	5.64 ± 0.39	5.73 ± 0.37*	5.74 ± 0.40*	5.76 ± 0.41*	<.001	<.001
HOMA-IR	1.11 ± 0.88	1.17 ± 0.78	1.20 ± 0.86	1.23 ± 0.82	.249	.003
Adipo-IR (µU/mL × mEq/L)	2317.2 ± 2322.2	2542.5 ± 2053.3	2807 ± 2297.0*	2989.6 ± 2449.7*	.001	<.001
Adiponectin (µg/mL)	12.20 ± 6.04	12.21 ± 6.44	13.33 ± 6.53	13.42 ± 7.47	.016	.016
C-reactive protein (ng/mL)	355.0 (170.0-763.5)	386.0 (215.0-933.5)	379.5 (190.8-801.0)	456.0 (232.0-833.0)	.465	.021
Insulin clearance (fasting)	0.35 ± 0.12	0.35 ± 0.23	0.34 ± 0.12	0.35 ± 0.15	.885	.753
Insulin clearance (AUC)	0.162 ± 0.046	0.160 ± 0.043	0.155 ± 0.047	0.153 ± 0.044	.046	.001
AUC-glucose during OGTT (mg·min/dL·10^4^)	1.73 ± 4.24	1.84 ± 3.93*	1.88 ± 4.16*	1.92 ± 4.27*	<.001	<.001
AUC-insulin during OGTT (µU·min/dL·10³)	5.29 ± 2.97	5.60 ± 3.32	6.13 ± 3.81*	6.10 ± 3.26*	.001	<.001
AUC–C-peptide during OGTT (ng·min/mL·10³)	0.76 ± 0.23	0.79 ± 0.24	0.82 ± 0.27*	0.83 ± 0.25*	.001	<.001
AUC–free fatty acids during OGTT (mEq·min/L·10^4^)	2.75 ± 1.10	2.98 ± 1.12*	3.10 ± 1.08*	3.42 ± 1.21^*,^*^†^*,*^‡^*	<.001	<.001

Data are expressed as n (%), means ± SD, or medians (interquartile range). *P* values are from a one-way analysis of variance or *χ^2^* test.

Abbreviations: Adipo-IR, adipose tissue insulin resistance index; AUC, area under the curve; HbA1c, glycated hemoglobin (hemoglobin A1c); HOMA-IR, homeostatic model assessment for insulin resistance; OGTT, oral glucose tolerance test.

^*^ < 0.05 vs age 65-69 group

^*†*^<0.05 vs age 70-74 group

^*‡*^<0.05 vs age 75-79 group with Bonferroni or Games–Howell correction.

FPG and fasting serum insulin levels were similar among the groups. However, HbA1c, AUC-glucose, and AUC-insulin were significantly higher in the 3 groups aged over 70 years than in the age 65 to 69 group. The Matsuda index was significantly lower in the age 80 to 84 group than in the age 65 to 69 group, suggesting a decrease in insulin sensitivity with age (Table 2). The insulin secretion index, calculated as AUC-insulin/AUC-glucose, and the insulinogenic index were comparable among the groups. The disposition index was significantly lower in the group aged 70 to 74 years and the group aged 80 to 84 years compared with the group aged 65 to 69 years, suggesting a decrease in β-cell function with age. Matsuda index and disposition index did not differ between groups older than 70 years, but the trend analysis showed that these decreased with age. Fasting FFA levels was significantly higher in the 3 groups aged over 70 years compared with the group aged 65 to 69 years. FFA levels were also higher in the 2 groups aged over 75 years than in the group aged 70 to 74 years. AUC-FFA during OGTT was also significantly higher in the 3 groups older than 70 years compared with the group aged 65 to 69 years and higher in the group aged 80 to 84 years than in groups younger than 80 years. Adipo-IR was significantly higher in the 2 groups aged over 75 years compared to the age 65 to 69 group. Adiponectin was slightly higher and AUC-insulin clearance was slightly lower in the older group, respectively. Trend analysis also showed similar age-related changes in these parameters.

**Table 2.** Metabolic parameters of the study participants

Age group (years)	65-69	70-74	75-79	80-84	*P* value	*P* for trend
AUC-insulin/AUC-glucose	0.31 ± 0.01	0.31 ± 0.01	0.33 ± 0.01	0.32 ± 0.02	.388	.251
Insulinogenic index	0.87 ± 0.09	0.68 ± 0.09	0.77 ± 0.10	0.72 ± 0.10	.133	.013
Matsuda index	8.18 ± 0.30	7.66 ± 0.30	7.58 ± 0.31	7.14 ± 0.32*	.013	<.001
Disposition index	2.21 ± 0.07	2.04 ± 0.07*	2.04 ± 0.07	1.99 ± 0.08*	.007	<.001

Data are expressed as means ± SE. *P* value for ANCOVA with adjustment for sex, hypertension, dyslipidemia, cardiovascular disease, and sarcopenia.

Abbreviation: AUC, area under the curve.

^*^ < 0.05 vs age 65-69 group

^*†*^<0.05 vs age 70-74 group

^*‡*^<0.05 vs age 75-79 group with Bonferroni or Games–Howell correction.

**Figure 1.**
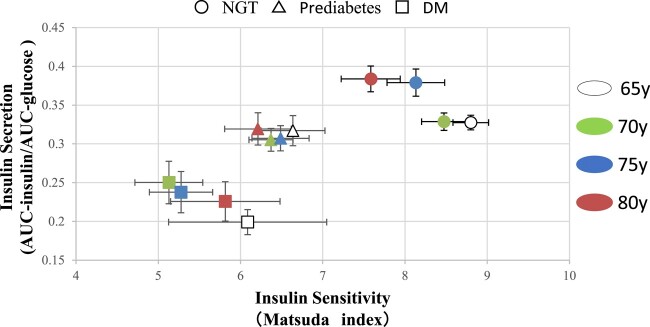
Relationship between insulin secretion (AUC-insulin/AUC-glucose) and sensitivity (Matsuda index) by glucose tolerance status and age group. Abbreviations: AUC, area under the curve; DM, diabetes mellitus; NGT, normal glucose tolerance.

Next, we evaluated the relationship between insulin secretion and insulin sensitivity with age based on glucose tolerance. We used the mean value and standard error of AUC-insulin/AUC-glucose and the Matsuda index in 3 groups (NGT, prediabetes, diabetes mellitus) (Fig. 1). In the NGT groups, insulin sensitivity seems to decline with age and higher insulin secretion associated with aging compensated for the decrease in insulin sensitivity. However, a clear relationship between insulin secretion or insulin sensitivity with age was not observed in participants with prediabetes or diabetes.

Since the Matsuda index and disposition index decreased with age, we tried to identify their determinants as the next step. We performed a simple correlation analysis between the Matsuda index or disposition index and various metabolic parameters related to fat accumulation (VFA, SFA, FFA, adiponectin, and CRP), muscle mass (SMI and ASM), muscle strength, and physical activity that have been previously reported to be associated with insulin action [22]. As shown in Table 3, the Matsuda index was significantly correlated with age, VFA, SFA, SMI, AMI, FFA, adiponectin and physical activity levels. The disposition index was significantly correlated with all of the parameters evaluated except for physical activity. VFA had the greatest correlation coefficient for both. In addition, SMI and ASM were negatively correlated with the Matsuda index and disposition index. Multiple regression revealed that VFA, SFA, and FFA were negatively correlated and adiponectin and physical activity were positively correlated with the Matsuda index. Similarly, VFA, SFA, and FFA were negatively correlated and adiponectin was positively correlated with the disposition index (Table 4). Among the parameters evaluated, VFA and FFA had a relatively high correlation with both the Matsuda index and disposition index. On the other hand, we were not able to find the independent correlation of ASM and handgrip strength with the Matsuda index or the disposition index.

**Table 3.** Results of simple correlation analysis between Matsuda index or disposition index and each parameter

	Matsuda index		Disposition index	
	Correlation coefficient	*P*	Correlation coefficient	*P*
Age	–0.121	<.001	–0.092	<.001
Sex	–0.030	.126	0.153	<.001
Visceral fat area	–0.438	<.001	–0.370	<.001
Subcutaneous fat area	–0.389	<.001	–0.204	<.001
Skeletal muscle mass index	–0.173	<.001	–0.249	<.001
Appendicular skeletal muscle mass	–0.105	<.001	–0.215	<.001
Handgrip strength	0.017	.523	–0.133	<.001
Free fatty acids	–0.246	<.001	–0.276	<.001
Adiponectin	0.286	<.001	0.272	<.001
C-reactive protein	–0.005	.420	–0.057	.030
Physical activity	0.094	<.001	0.032	.231

**Table 4.** Multiple regression analysis for the Matsuda index or disposition index

Matsuda index					
Model		B	SE	β	*P*
1	Constant	16.845	1.328		<.001
	Age	–0.048	0.018	–.063	.007
	Sex	–1.242	0.201	–.149	<.001
	Visceral fat area	–0.051	0.003	–.471	<.001
2	Constant	18.325	2.192		<.001
	Age	−0.052	0.019	−.068	.006
	Sex	−1.035	0.394	−.124	.009
	Visceral fat area	−0.030	0.003	−.272	<.001
	Subcutaneous fat area	−0.013	0.002	−.179	<.001
	Appendicular skeletal muscle mass	−0.044	0.048	−.044	.363
	Handgrip Strength	−0.007	0.024	−.012	.775
	Free fatty acids	−0.004	0.000	−.198	<.001
	Adiponectin	0.120	0.016	.190	<.001
	C-reactive protein	1.601·10^−5^	0.000	.023	.309
	Physical activity	0.004	0.002	.047	.034
Disposition index					
1	Constant	3.103	0.324		<.001
	Age	–0.009	0.004	–.052	.033
	Sex	0.138	0.049	.071	.005
	Visceral fat area	–0.009	0.001	–.343	<.001
2	Constant	3.924	0.532		<.001
	Age	−0.008	0.005	−.048	.067
	Sex	0.035	0.096	.018	.715
	Visceral fat area	−0.005	0.001	−.209	<.001
	Subcutaneous fat area	−0.001	0.000	−.072	.019
	Appendicular skeletal muscle mass	−0.015	0.012	−.063	.211
	Handgrip Strength	−0.006	0.006	−.043	.327
	Free fatty acids	−0.001	0.000	−.275	<.001
	Adiponectin	0.022	0.004	.154	<.001
	C-reactive protein	−5.043·10^−6^	0.000	−.031	.187
	Physical activity	0.000	0.000	.008	.725

Abbreviations: B, the unstandardized β; SE, standard error; β, standardized β.

## Discussion

The purpose of this study was to investigate whether insulin sensitivity and β-cell function deteriorate after the age of 65 years and the factors contributing to the exacerbation of glucose tolerance with aging among older adults with no history of diabetes. The present study showed that the prevalence of newly diagnosed diabetes was higher in the older group. The Matsuda index and disposition index decreased with age, suggesting that the increased prevalence may be due to decreased insulin sensitivity and β-cell function. In addition, multiple regression revealed that both the Matsuda index and disposition index were strongly correlated with VFA and FFA; thus, FFA level in blood and its source could be a determinant.

Previous studies have reported that lower insulin secretion index and impaired insulin sensitivity in older adults as compared with younger individuals contribute to impaired glucose tolerance in older adults [4, 6]. However, it remains unclear whether these trends are further exacerbated by aging beyond age 65. Our findings suggested that the ability to secrete insulin in response to blood glucose levels, reflected by the insulinogenic index and AUC-insulin/AUC-glucose, does not worsen after the age of 65 years, but insulin sensitivity (Matsuda index) and disposition index showed age-related declines. Our results differ from a previous report that demonstrated that the insulinogenic index of older adults was lower than younger adults [4]. Taken together, these data indicate that the age-related decrease in the insulinogenic index may be slower in older adults. However, it could also be possible that these indices are affected by features of present study subjects. Therefore, further study is needed in this regard. The disposition index is useful for assessing an individual's ability to compensate for changes in insulin secretion by adjusting insulin sensitivity; it is considered to be more appropriate to evaluate the accuracy of β-cell function [15]. In fact, as shown in Fig. 1, only individuals with normal glucose tolerance were able to secrete insulin to compensate for age-related insulin resistance, while individuals with prediabetes or type 2 diabetes had more insulin resistance but failed to secrete insulin to compensate for it.

In the present study, we observed an exacerbation of insulin resistance with increasing age, which was positively correlated with increasing SFA, VFA, and FFA but negatively correlated with adiponectin. Similarly, it has been suggested that increased FFA and Adipo-IR and decreased adiponectin occur with increased body fat, both of which promote ectopic fat accumulation, leading to insulin resistance and metabolic disorders [23, 24]. In addition, it has also been suggested that SFA and VFA are major sources of whole-body FFA release [25-28]. In the present study, FFA is only weakly correlated with VFA (*r* = 0.06, *P* = .024). The results of multiple regression analysis also suggest that FFA is a factor independent of VFA in the Matsuda index. In addition, FFA showed a significant positive correlation with VFA only in the 65 to 69 age group, but not in the groups aged over 70 years (data were not shown). On the other hand, a significant negative correlation was found between FFA and ASM, the latter is inversely related to total body fat, in the age groups over 70 years (data were not shown). This suggests that total body fat mass, rather than VFA, may be a major determinant of FFA in older adults. There was little change in adiponectin levels or SFA, although there was an increase in VFA and FFA with aging. These results suggest that age-related increases in VFA and FFA might be responsible for the exacerbation of insulin resistance with aging. On the other hand, VFA and adiponectin showed a significant negative correlation (*r* = −0.42, *P* < .001). This suggests that adiponectin is not a factor that declines with age, although it does affect insulin resistance.

Multiple regression revealed that FFA is the most relevant variable for the disposition index. When FFA concentrations are chronically elevated, a variety of changes occur in β cells, such as increased endoplasmic reticulum stress, increased oxidative stress, more inflammation, and increased autophagy flux [29]. Although these changes are compensatory mechanisms necessary for β-cell survival, in β cells of individuals susceptible to diabetes, these stresses might result in decreased insulin secretion and increased apoptosis, leading to impaired glucose tolerance.

Decrease in skeletal muscle mass [30] and strength [31, 32] is associated with insulin resistance in older adults. While we observed a simple correlation between skeletal muscle mass, the largest uptake organ of glucose, and both the Matsuda index and disposition index, multiple regression highlighted body fat mass, rather than skeletal muscle mass, as a significant contributor to the Matsuda index and disposition index. This suggests that a reduction in skeletal muscle mass might exert a less direct influence on insulin sensitivity or β-cell function in older adults. Given the inverse relationship between body fat mass and skeletal muscle mass, skeletal muscle mass might primarily serve as a confounding factor in this context. Similarly, handgrip strength showed no correlation with Matsuda index. Although it showed a single correlation with disposition index, the results of multiple regression analysis showed no independent association in this study. On the other hand, low handgrip strength is associated with insulin resistance in patients with type 2 diabetes [33], and low handgrip strength has been shown to be an independent risk factor for the development of type 2 diabetes [34]. These data suggest that while low handgrip strength is a risk factor for type 2 diabetes, low handgrip strength is not linked to insulin resistance before the onset of diabetes.

In terms of inflammation pathways, chronic inflammation markers such as high-sensitivity CRP, IL-6, and TNF-α have been shown to be elevated prior to the onset of diabetes [35]. On the other hand, a prospective study investigating the risk of developing type 2 diabetes reported no significant association between high-sensitivity CRP and the development of diabetes when adjusted for other factors such as BMI [36]. The association between CRP and insulin resistance may be an epiphenomenon of obesity or adiposity, rather than an independent factor, and, in fact, a study of Korean subjects showed that slight weight gain (BMI ≥23 kg/m^2^) was a greater risk for insulin resistance than high CRP levels [37]. Thus, in this study, we included CRP as a factor to adjust multiple regression analysis for the Matsuda index and disposition index (Table 4), although CRP and Matsuda index and disposition index did not show independent associations in this study.

Postprandial hyperglycemia is a common characteristic of glucose intolerance in older adults [38]. In the present study, although HbA1c, AUC-glucose, and AUC-insulin increased with age, there were no differences in fasting glucose or insulin levels among the groups. The incidence of type 2 diabetes is significantly higher in older adults than in younger people. Periodic and appropriate screening is essential for early diagnosis and proper treatment. Thus, assessing postprandial glucose levels, rather than fasting levels, appears to be more important in screening for type 2 diabetes in older adults.

There are several limitations in this study. First, because of the cross-sectional design, it was not possible to track changes in insulin secretion or insulin resistance over time for each individual. Further observational studies are intended to clarify these issues. Second, as age increased, participants might have been healthier, potentially introducing a survival bias. The group aged over 80 years had the highest energy intake, which might be related to this bias. If so, this high-calorie, high-carbohydrate diet may contribute to the deterioration of glucose tolerance. Thus, the older the age group, the more caution should be exercised in interpreting the results. Third, because the study population consisted of older adults who were living in central Tokyo and had a higher level of education, caution is required when generalizing our findings to other populations. Finally, since participants younger than 65 years were not included, these results are not applicable to those under 65 years of age.

In conclusion, this study revealed that glucose tolerance declines with age in older Japanese adults aged 65 years or older, potentially due to insulin resistance and decreased β-cell function, at least in part caused by age-related fat accumulation and elevated FFA levels. To address these problems and prevent the pandemic of new-onset diabetes in older adults, improvements in body composition through appropriate diet and exercise might be effective to counter exacerbation of glucose tolerance, even in older adults. In addition, regular measurement of body composition and postprandial blood glucose levels, as well as body weight and fasting blood glucose levels, may be useful in early identification of these changes.

## Data Availability

Some or all datasets generated during and/or analyzed during the current study are not publicly available but are available from the corresponding author on reasonable request.

## Clinical Trial Registration

The study protocol was approved by the ethics committee of Juntendo University in November 2015 (Nos. 2015078, and M15-0057).

## Abbreviations

Adipo-IR: adipose tissue insulin resistance index
ASM: appendicular skeletal muscle mass
AUC: area under the curve
CRP: C-reactive protein
DXA: dual-energy x-ray absorptiometry
FFA: free fatty acid
FPG: fasting plasma glucose
HbA1c: glycated hemoglobin (hemoglobin A1c)
HOMA-IR: homeostatic model assessment for insulin resistance
MRI: magnetic resonance imaging
NGT: normal glucose tolerance
OGTT: oral glucose tolerance test
SFA: subcutaneous fat area
SMI: skeletal muscle mass index
VFA: visceral fat area
